# Associations of tear mediators with ocular surface symptoms, signs, and corneal nerve morphology in dry eye disease: a cross-sectional study

**DOI:** 10.3389/fmed.2026.1929287

**Published:** 2026-09-04

**Authors:** Lijin Cui, Ruixuan Jia, Chenyue Yu, Zhaoxiang Xu, Yao Yao, Jian Guo, Xiaotong Ren

**Affiliations:** 1 Department of Ophthalmology, The First Affiliated Hospital of Fujian Medical University, Fuzhou, Fujian, China; 2 Department of Ophthalmology, National Regional Medical Center, Binhai Campus of the First Affiliated Hospital, Fujian Medical University, Fuzhou, Fujian, China; 3 Fujian Provincial Clinical Medical Research Center of Eye Diseases and Optometry, The First Affiliated Hospital of Fujian Medical University, Fuzhou, Fujian, China; 4 Nanping Wuyi Housheng Eye Hospital, Nanping, China

**Keywords:** corneal nerves, dry eye disease (DED), microneuroma, MMP-9, tear mediators, β-endorphin

## Abstract

**Purpose:**

To investigate associations between tear mediators and ocular symptoms, signs, and corneal nerve parameters in patients with dry eye disease (DED).

**Methods:**

This cross-sectional study included 101 DED patients. Six tear mediators (IL-6, NGF, SP, CGRP, MMP-9, β-endorphin) were measured by Luminex. Symptoms (10-item questionnaire and OSDI), signs (TMH, TBUT, CFS), and corneal subbasal nerve parameters were assessed. Spearman's correlation was used. For eye-specific parameters, values from both eyes were averaged to obtain patient-level measurements.

**Results:**

β-Endorphin showed a strong negative correlation with ocular pain (*r* = −0.813, *p* < 0.001) and a positive correlation with corneal nerve fiber length (*r* = 0.722, *p* = 0.030). Microneuroma count correlated positively with IL-6 (*r* = 0.285), SP (*r* = 0.294), and MMP-9 (*r* = 0.376), and negatively with β-endorphin (*r* = −0.212, all *p* < 0.05). MMP-9, NGF, and SP strongly correlated with OSDI (*r* = 0.907, 0.901, 0.894, respectively, all *p* < 0.001). All tear mediators except β-endorphin correlated with CFS, but none correlated with TMH or TBUT.

**Conclusion:**

Tear β-endorphin is uniquely associated with less pain and better corneal nerve preservation. Microneuromas link to pro-inflammatory mediators. Tear mediators reflect ocular surface damage (CFS) but not tear quantity and stability, suggesting that tear mediators profiling may reflect inflammatory and neuropathic mechanisms in DED, warranting further validation in controlled studies.

## 1 Introduction

Dry eye disease (DED) is a multifactorial disorder characterized by tear film instability, hyperosmolarity, inflammation, and neurosensory abnormalities (1). According to TFOS DEWS III (2025), DED is defined as “a multifactorial, symptomatic disease” emphasizing that DED is always symptomatic and neural dysfunction is a core component (2). DED affects 5%–50% of the global population, with higher prevalence in Asia (3, 4), and significantly impairs quality of life and work productivity (5, 6).

A major challenge in DED management is the discordance between symptoms and signs (7). Many patients report debilitating symptoms that correlate poorly with objective measures such as tear break-up time (BUT) (8). DEWS III explicitly recognizes that patient-reported symptoms are essential for diagnosis (2), and neurosensory abnormalities are increasingly recognized as key contributors to this discordance (3, 9).

Inflammation plays a central role in DED (10, 11). Pro-inflammatory mediators and neuropeptides are elevated in tears and contribute to tissue damage and symptom generation (12, 13). IL-6 correlates with disease severity and corneal staining (14, 15). Matrix metalloproteinase-9 (MMP-9) has been proposed as a point-of-care biomarker, and recent studies showed clinical phenotypes can predict positive MMP-9 tests (16). Neuropeptides such as substance P (SP) and calcitonin gene-related peptide (CGRP) are involved in neurogenic inflammation and pain (17, 18). A 2025 longitudinal study found that tear SP increases over time, suggesting persistent neurogenic inflammation (19). Tear nerve growth factor (NGF) levels are increased in DED and correlate with disease severity (20, 21).

β-endorphin, an endogenous opioid peptide with potent analgesic properties, but its role in ocular pain remains underexplored (22, 23). Yang et al. measured tear β-endorphin in DED but did not report correlation with pain severity (24). Given DEWS III’ s emphasis on neural dysfunction (2), understanding endogenous pain modulation in DED is critical.

This cross-sectional study aimed to investigate associations between tear mediators (IL-6, NGF, SP, CGRP, MMP-9, β-endorphin) and specific ocular symptoms and signs in DED patients, investigating their associations with specific symptoms and signs in DED patients.

## 2 Methods

### 2.1 Study design and participants

This cross-sectional study included 101 patients diagnosed with DED, which was approved by the Ethics Committee of the First Affiliated Hospital of Fujian Medical University (ethics approval No. MTCA, ECFAH of FMU [2015]084-2) and adhered to the principles of the Declaration of Helsinki. All participants provided written informed consent.

Patients were recruited from the Ophthalmology Department of the First Affiliated Hospital of Fujian Medical University between October 2024 and March 2025. Inclusion criteria were: (1) age 18–75 years; (2) diagnosis of DED according to TFOS DEWS II criteria (1), which required: (a) OSDI score ≥ 13, (b) TBUT ≤ 5 seconds or Schirmer I test ≤ 5 mm/5 min, and (c) corneal fluorescein staining score ≥ 1. Patients with any other ocular or systemic condition that could affect tear film or corneal nerves were excluded. Exclusion criteria included: other corneal diseases, glaucoma, uveitis, retinal disorders, systemic diseases known to affect corneal nerves (diabetes, Parkinson's disease, trigeminal palsy, vitamin A deficiency), recent ocular surgery within 3 months, use of punctal plugs, recent meibomian gland expression or photothermal therapy, use of cyclosporine or ocular steroids, contact lens wear, skin disorders, and pregnancy or lactation.

### 2.2 Symptom assessment

All patients completed two symptom questionnaires.

Ten-symptom questionnaire: This instrument was developed by our research team and has been used in previous studies (25, 26), where it demonstrated meaningful clinical associations with corneal nerve parameters and symptom improvement following treatment. Although it has not undergone formal psychometric validation in a dedicated cohort, the instrument has demonstrated clinical relevance in the DED population through its ability to detect symptom- specific associations with objective parameters and treatment responses in our previous studies. It covers 10 DED-related symptoms: dryness, foreign body sensation, pain, burning, watering, asthenopia, blurred vision, itching, increased secretions, and photophobia. Each symptom was scored on a visual analog scale from 0 (“not at all”) to 10 (“very severe”). The sum of the 10 scores was calculated as the total symptom score.

Ocular Surface Disease Index (OSDI): The Chinese version of the OSDI questionnaire was used to assess symptom severity over the past week (27). The OSDI consists of 12 items covering ocular symptoms, visual function, and environmental triggers. Scores range from 0 to 100, with higher scores indicating more severe symptoms. Severity categories were defined as: normal (0–12), mild (13–22), moderate (23–32), and severe (33–100) (26, 27).

### 2.3 Ocular sign assessment

Tear meniscus height (TMH): TMH was measured using an Oculus Keratograph 5M (Oculus, Wetzlar, Germany) under white light illumination without fluorescein. Each eye was measured three times, and the average value was recorded. Artificial intelligence-assisted methods have been developed to improve the objectivity and reproducibility of TMH measurements in multicenter settings (28).

Tear break-up time (TBUT): Fluorescein sodium strips (Tianjin Jingming New Technology Development Co., Ltd., China) were moistened with 0.9% saline and applied to the inferior conjunctival fornix without contacting the cornea. Patients were instructed to blink gently and then keep eyes open. The time from the last blink to the appearance of the first black spot on the cornea was recorded using a slit lamp with cobalt blue filter. Each eye was measured three times, and the average value was recorded.

Corneal fluorescein staining (CFS): Corneal staining was assessed using the van Bijsterveld grading system (29). The cornea was divided into four quadrants, each scored 0 (no staining), 1 (1–6 staining spots), 2 (7–30 spots), or 3 (>30 spots). The total score ranged from 0 to 12.

Expressibility and secretion quality were assessed separately for the upper and lower eyelids using the scoring systems described above. For statistical analysis, the scores from the upper and lower eyelids were averaged to obtain a single patient-level measure of meibomian gland function.

### 2.4 *In vivo* confocal microscopy (IVCM) assessment

Corneal subbasal nerve parameters were assessed using a digital corneal confocal laser scanning microscope (Heidelberg Retina Tomograph II with Rostock Cornea Module, Heidelberg Engineering GmbH, Germany). After topical anesthesia with 0.4% oxybuprocaine hydrochloride (Benoxil, Santen Pharmaceutical Co., Japan), a drop of 0.2% carbomer (Vidisic, Dr. Gerhard Mann Chemical-Pharmaceutical Co., Germany) was applied as a coupling agent. The patient's chin was placed on the chin rest with the forehead against the headband. Using the “Section” mode, all IVCM images were acquired from the central cornea, with at least 200 non-overlapping images captured per eye. For each eye, the subbasal nerve plexus was identified by the presence of hyperreflective nerve fibers running parallel to the epithelial basal cell layer.

Three images with optimal nerve visibility were selected for analysis by a single experienced examiner (X.R.) who was masked to patients’ clinical data and tear mediator profiles, following a predefined standardized protocol. The selection criteria were: (1) optimal focus — nerve fibers appeared sharply delineated without blurring; (2) maximal nerve coverage — the image contained the greatest number of visible nerve fibers within the frame; and (3) absence of artifacts — images with blink artifacts, folds, shadows, or debris were excluded. This protocol has been used in our previous studies (25, 26).

Corneal subbasal nerve parameters were quantified using ImageJ software (National Institutes of Health, USA) as previously described (25, 26). The following parameters were quantified: corneal nerve fiber length (CNFL, total length of subbasal nerves per frame, converted to nerve density in mm/mm^2^ based on a frame area of 0.16 mm^2^ [400 × 400 μm]), corneal nerve fiber width (CNFW, mean diameter of nerve fibers,*μ*m), corneal nerve fiber reflectivity (CNFR, mean grayscale of nerve fibers, grey values), and microneuroma count (number of bead-like terminal enlargements ≥ 5 μm in diameter per frame). The average values from three images were recorded for each eye.

### 2.5 Tear collection and mediators measurement

Non-stimulated basal tear fluid was collected separately from each eye from the inferior tear meniscus using calibrated glass microcapillary tubes (Hirschmann, Germany) in a non-traumatic manner, avoiding touching the conjunctiva or cornea to prevent reflex tearing as much as possible. The capillary tube was placed at the tear meniscus, and tear fluid was drawn by capillary action. Approximately 5–10 μL of tear fluid was collected from each eye. Immediately after collection, the tear sample was diluted in ice-cold Sample Diluent to a final volume of 50 μL in a 0.2-mL Eppendorf tube (Axygen, USA) and stored at −80°C until analysis. If the initial collection was insufficient, repeated collections were performed with care to avoid reflex tearing.

Tear samples from each eye were processed and measured independently using the Luminex assay. The concentrations obtained from the right and left eyes were then averaged to generate a single patient-level value for each mediator, consistent with the averaging of bilateral ocular parameters (TMH, TBUT, CFS, and IVCM parameters) used throughout this study. Six tear mediators — IL-6, NGF, SP, CGRP, MMP-9, and β-endorphin — were measured using a Luminex multiplex assay on a Luminex 200TM instrument (Luminex Corporation, Austin, TX, USA) with reagents from R&D Systems (30). All samples were assayed in duplicate in a single batch according to the manufacturer's protocols. Standard curves were generated using the provided standards, and sample concentrations were interpolated from the standard curves. For the Luminex assay, 50 μL of the diluted sample (representing approximately 5–10 μL of neat tear fluid) was used per well. Samples with concentrations below the lower limit of detection as flagged by the instrument software were assigned a value of half the respective limit for statistical analysis (30). Mediator concentrations were not normalized to total tear protein.

### 2.6 Statistical analysis

Statistical analyses were performed using SPSS version 26.0 (SPSS Inc., Chicago, IL, USA). Normality of continuous variables was assessed using the Kolmogorov–Smirnov test. Descriptive data are presented as mean ± standard deviation (SD). To account for inter-eye correlation, all eye-specific parameters (TMH, TBUT, CFS, and corneal nerve parameters) were averaged across both eyes to obtain a single patient-level measurement for each participant. All correlation analyses were therefore performed on patient-level data (*n* = 101).

Since tear mediator levels and symptom scores were not normally distributed, Spearman’ s rank correlation coefficient was used to assess correlations between mediator levels and clinical parameters (symptom scores, OSDI, TMH, TBUT, CFS, and corneal nerve parameters). Correlation coefficients (r) were interpreted as weak (0.1–0.3), moderate (0.3–0.7), or strong (≥0.7). Given the exploratory nature of this study, we primarily report uncorrected Spearman correlation coefficients with their corresponding *p*-values. To assist readers in assessing the robustness of the findings, correlations that remained statistically significant after Benjamini–Hochberg false discovery rate (FDR) correction (*q* < 0.05) are marked with an asterisk (*) in the tables. This approach balances the risk of type I errors (false positives) due to multiple comparisons against the risk of type II errors (false negatives) that may arise from over-adjustment in an exploratory study, potentially obscuring clinically meaningful signals that warrant further investigation. All reported correlations were based on complete data from all 101 participants, as there were no missing values for any of the tear mediators, symptom scores, or clinical parameters included in the analyses.

Given the relatively small sample size, we did not perform multivariable adjustment for potential confounders (e.g., age, sex, DED subtype). These factors are acknowledged as limitations in the Discussion.

## 3 Results

### 3.1 Participant characteristics

A total of 101 DED patients (202 eyes) were included in this cross-sectional analysis. All subsequent correlation analyses were performed on patient-level averaged data (*n* = 101). The mean age of participants was 52.36 ± 16.02 years (range 21–72 years). Females comprised 60.4% (61/101) of the cohort. The mean OSDI score was 48.85 ± 16.33, indicating moderate-to-severe DED. Baseline characteristics are summarized in Table 1.

**Table 1:** General information of the patients.

General information	Results
Number (People)	101
Number (Eyes)	202
Sex (Male/female)	40/61
Age(Years, Mean ± SD, Range)	52.36 ± 16.02 (21–72)
OSDI (Scores, Mean ± SD, Range)	48.85 ± 16.33 (13–80)

### 3.2 Correlation between tear mediators and ocular symptoms

The correlations between the six tear mediators and DED symptoms are presented in Table 2.

**Table 2:** Correlations between tear mediators and ocular symptoms.

Symptoms		IL-6	NGF	SP	CGRP	MMP-9	β-endorphin
Dryness	r	−0.091	−0.183	−0.086	0.195	0.015	0.021
	P	0.491	0.223	0.417	0.204	0.888	0.856
Foreign body sensation	r	0.397**	−0.142	−0.092	−0.047	0.102	−0.104
	P	0.002	0.346	0.384	0.762	0.327	0.373
Pain	r	0.118	−0.233	0.032	0.047	−0.009	−0.813**
	P	0.368	0.120	0.759	0.763	0.932	<0.001
Burning	r	0.061	−0.074	0.025	0.242	0.035	−0.176
	P	0.646	0.627	0.816	0.113	0.734	0.128
Watering	r	0.305*	−0.098	0.013	−0.090	−0.084	0.000
	P	0.018	0.571	0.901	0.561	0.419	0.998
Asthenopia	r	0.062	−0.278	0.032	−0.095	0.077	−0.132
	P	0.636	0.061	0.765	0.542	0.459	0.255
Blurred vision	r	−0.208	−0.049	0.273**	−0.042	0.145	0.215
	P	0.111	0.747	0.009	0.787	0.163	0.063
Itching	r	0.265*	−0.260	−0.118	−0.207	0.021	−0.040
	P	0.041	0.080	0.264	0.179	0.842	0.729
Increased secretions	r	0.455**	−0.215	−0.122	−0.182	−0.092	0.159
	P	<0.001	0.152	0.245	0.236	0.380	0.170
Photophobia	r	0.181	0.083	0.097	0.021	0.125	−0.053
	P	0.167	0.582	0.358	0.894	0.231	0.648
Total	r	0.380**	−0.266	0.010	−0.035	0.094	−0.138
	P	0.003	0.074	0.923	0.823	0.369	0.236
OSDI	r	0.811**	0.901**	0.894**	−0.697**	0.907**	−0.098
	P	<0.001	<0.001	<0.001	<0.001	<0.001	0.401

Correlations marked with * or ** remained statistically significant after Benjamini–Hochberg false discovery rate correction (*q* < 0.05). All correlation analyses were performed on complete data (*n* = 101 for every reported correlation).

NGF, nerve growth factor; SP, substance P; CGRP, calcitonin gene-related peptide; MMP-9, matrix metalloproteinase-9; r, correlation coefficient.

* *P*<0.05.

** *P*<0.01.

IL-6 showed moderate to strong positive correlations with multiple sensory symptoms. Specifically, IL-6 was positively correlated with foreign body sensation (*r* = 0.397, *P* = 0.002), watering (*r* = 0.305, *P* = 0.018), itching (*r* = 0.265, *P* = 0.041), increased secretions (*r* = 0.455, *P* < 0.001), and total symptom score (*r* = 0.380, *P* = 0.003). Notably, IL-6 demonstrated a very strong positive correlation with OSDI (*r* = 0.811, *P* = 0.001).

NGF showed a remarkably strong positive correlation with OSDI (*r* = 0.901, *P* < 0.001), but did not significantly correlate with individual symptom scores. This suggests that NGF may reflect overall disease severity rather than specific symptom types.

SP was positively correlated with blurred vision (*r* = 0.273, *P* = 0.009) and showed a very strong positive correlation with OSDI (*r* = 0.894, *P* < 0.001).

CGRP demonstrated a strong negative correlation with OSDI (*r* = −0.697, *P* < 0.001), indicating that lower CGRP levels are associated with more severe disease. No significant correlations were found between CGRP and individual symptom scores.

MMP-9 showed the strongest positive correlation with OSDI among all six mediators (*r* = 0.907, *P* < 0.001), confirming its role as a robust biomarker of DED severity. The total symptom score, calculated as the unweighted sum of 10 individual symptom intensities, may not fully capture the multidimensional nature of symptom burden as measured by the validated OSDI. Therefore, the absence of significant correlations with the total score should be interpreted with caution.

β-endorphin was the only mediator that correlated specifically with ocular pain. It showed a strong negative correlation with pain symptom score (*r* = −0.813, *P* < 0.001), suggesting that higher tear β-endorphin levels are associated with lower pain intensity. No significant correlation was found between β-endorphin and OSDI (*P* = 0.401).

No significant correlations were observed between any mediators and the symptoms of dryness, burning, or photophobia.

The correlation between OSDI and the unweighted total symptom score was *r* = 0.541 (*P* < 0.001), indicating that while these two instruments are moderately to strongly correlated, they capture related but distinct constructs.

To visually assess the robustness of the strongest correlations, scatter plots for the key associations (MMP-9, NGF, SP, IL-6 vs. OSDI, and β-endorphin vs. pain) are provided in Supplementary Figures S1–S5.

### 3.3 Correlation between tear mediators and ocular signs

The correlations between tear mediators and ocular signs are summarized in Table 3.

**Table 3:** Correlations between tear mediators and ocular signs.

Signs		IL-6	NGF	SP	CGRP	MMP-9	β-endorphin
TMH(mm)	r	0.062	0.161	0.050	−0.204	0.077	0.180
	P	0.637	0.286	0.639	0.185	0.462	0.119
TBUT(s)	r	0.030	0.186	0.131	−0.209	0.017	0.016
	P	0.821	0.215	0.213	0.174	0.871	0.894
CFS	r	0.349**	0.701**	0.711**	−0.571**	0.703**	0.030
	P	0.006	<0.001	<0.001	<0.001	<0.001	0.795
Expressibility	r	0.140	0.014	0.061	0.108	0.047	0.224
	P	0.285	0.927	0.561	0.486	0.651	0.052
Secretion quality	r	−0.043	−0.213	−0.168	0.397**	−0.160	0.119
	P	0.746	0.156	0.110	0.008	0.123	0.306

Correlations marked with * or ** remained statistically significant after Benjamini–Hochberg false discovery rate correction (*q* < 0.05). All correlation analyses were performed on complete data (*n* = 101 for every reported correlation).

TMH: tear meniscus height; TBUT: tear film break-up time; CFS: corneal fluorescein staining; NGF: nerve growth factor; SP: substance P; CGRP: calcitonin gene-related peptide; MMP-9: matrix metalloproteinase-9; r: correlation coefficient.

** *P*<0.01.

CFS showed significant correlations with multiple mediators. IL-6 (*r* = 0.349, *P* = 0.006), NGF (*r* = 0.701, *P* < 0.001), SP (*r* = 0.711, *P* < 0.001), and MMP-9 (*r* = 0.703, *P* < 0.001) all demonstrated positive correlations with CFS. CGRP showed a significant negative correlation with CFS (*r* = −0.571, *P* < 0.001). β-endorphin showed no significant correlation with CFS (*P* = 0.795).

In contrast, none of the six tear mediators showed significant correlations with TMH or TBUT. This finding suggests that tear mediator levels more closely reflect ocular surface damage (as measured by CFS) rather than tear quantity (TMH) or stability (TBUT).

### 3.4 Correlation between tear mediators and corneal nerve parameters

The correlations between tear mediators and corneal subbasal nerve parameters are presented in Table 4.

**Table 4:** Correlations between tear mediators and corneal nerve parameters.

Tear mediators		CNFL	CNFW	CNFR	Microneuroma
IL-6	r	0.068	0.163	−0.166	0.285*
	P	0.062	0.316	0.222	0.027
NGF	r	0.087	−0.070	−0.106	−0.028
	P	0.634	0.713	0.484	0.852
SP	r	−0.084	0.037	−0.093	0.294*
	P	0.566	0.793	0.390	0.004
CGRP	r	0.182	−0.124	0.023	−0.011
	P	0.250	0.528	0.884	0.942
MMP-9	r	−0.096	0.117	−0.047	0.376**
	P	0.372	0.413	0.665	＜0.001
β-endorphin	r	0.722*	−0.299	−0.062	−0.212*
	P	0.030	0.051	0.610	0.027

Correlations marked with * or ** remained statistically significant after Benjamini–Hochberg false discovery rate correction (*q* < 0.05). All correlation analyses were performed on complete data (*n* = 101 for every reported correlation).

CNFL, Corneal nerve fiber length; CNFW, Corneal nerve fiber width; CNFR, Corneal nerve fiber reflectivity; NGF, nerve growth factor; SP, substance P; CGRP, calcitonin gene-related peptide; MMP-9, matrix metalloproteinase-9; r, correlation coefficient.

* *P*<0.05.

** *P*<0.01.

Corneal nerve fiber length showed a significant positive correlation with tear β-endorphin levels (*r* = 0.722, *P* = 0.030), indicating that patients with longer corneal nerves had higher β-endorphin levels. No significant correlations were found between nerve fiber length and other mediators (IL-6, NGF, SP, CGRP, MMP-9). Corneal nerve width and nerve reflectivity showed no significant correlations with any of the six tear mediators.

Microneuroma count demonstrated significant positive correlation with IL-6 (*r* = 0.285, *P* = 0.027), SP (*r* = 0.294, *P* = 0.004), MMP-9 (*r* = 0.376, *P* < 0.001), and negative correlation with β-endorphin (*r* = −0.212, *P* = 0.027). These findings suggest that a higher number of microneuromas—a marker of aberrant nerve regeneration—is associated with a pro-inflammatory mediator profile (elevated IL-6, SP, MMP-9) and lower levels of the analgesic peptide β-endorphin.

## 4 Discussion

This cross-sectional study investigated the associations between tear mediator levels and ocular symptoms and signs in 101 DED patients. The key findings are: (1) β-endorphin showed a strong negative correlation with ocular pain, representing the first report of this specific association in DED; (2) MMP-9, NGF, and SP demonstrated very strong positive correlations with OSDI, supporting their role as biomarkers of overall disease severity; (3) IL-6 correlated with multiple sensory symptoms including foreign body sensation, itching, and secretion; (4) CGRP showed a negative correlation with OSDI and CFS; and (5) none of the tear mediators correlated with TMH or TBUT, whereas all except β-endorphin correlated with CFS, suggesting they better reflect ocular surface damage than tear quantity or stability.

### 4.1 β-endorphin and ocular pain

The most novel finding of this study is the strong negative correlation between tear β-endorphin levels and ocular pain (*r* =  −0.813, *P* < 0.001). β-endorphin is an endogenous opioid peptide with potent analgesic properties, known to increase pain thresholds through activation of μ-opioid receptors (31). In DED, chronic nociceptive input from corneal nerve injury can lead to peripheral and central sensitization, resulting in neuropathic ocular pain that may persist even in the absence of ongoing tissue damage (22, 32). The present finding suggests that endogenous analgesic mechanisms involving β-endorphin may be activated in DED patients to counteract pain. This is consistent with the emerging concept that ocular pain is modulated by endogenous opioid peptides, including enkephalins, endorphins, and dynorphins (22, 23).

Notably, Yang et al. previously measured tear β-endorphin in DED patients and found similar levels between DED and control groups, and did not report a correlation between β-endorphin and pain (24). There are several possible explanations for the discrepancy. First, our cohort had higher baseline OSDI scores (mean 48.85, indicating moderate-to-severe DED), whereas Yang et al. included patients with more variable severity. Second, we specifically examined the correlation with pain severity using a 10-point visual analog scale, whereas the previous study focused on group comparisons of neuropeptide levels. Third, our larger sample size (101 patients vs. 38 DED patients) provided greater statistical power to detect this association. Our results suggest that β-endorphin's role in pain modulation may only become apparent in more severe disease or when pain is assessed as a continuous measure rather than a binary presence/absence. This finding aligns with the broader concept that endogenous pain modulation systems are recruited in response to persistent nociceptive input, as has been observed in other chronic pain conditions (22, 31).

### 4.2 MMP-9 and disease severity

MMP-9 showed the strongest positive correlation with OSDI among all tear mediators examined (CC = 0.907, *p* < 0.001). This finding aligns with the established role of MMP-9 as a key mediator of ocular surface inflammation in DED. MMP-9 degrades the corneal epithelial basement membrane, tight junction proteins, and occludins, thereby disrupting epithelial barrier function (15, 33). Higher tear MMP-9 has been reported to have 85% sensitivity and 95% specificity for DED diagnosis, and point-of-care tests (e.g., InflammaDry) are now available for clinical use (34, 35). Of note, while MMP-9 showed a strong correlation with the OSDI—a validated, weighted questionnaire—it did not correlate with the unweighted sum of individual symptom scores (Total symptom score, *r* = 0.094, *P* = 0.369). This discrepancy suggests that the relationship between MMP-9 and symptom perception is better captured by the multidimensional structure of the OSDI than by a simple arithmetic sum of symptom intensities, and warrants further investigation in future studies.

A recent 2025 study by Mejía-Salgado et al. evaluated 1,363 DED patients and identified specific clinical phenotypes that predict positive InflammaDry results, including corneal staining (Oxford ≥3, OR = 2.41), conjunctival staining (Oxford ≥3, OR = 2.30), and autoimmune or allergic disease (OR = 1.59) (16). Our results extend these findings by demonstrating a very strong correlation between MMP-9 levels and symptom severity, suggesting that MMP-9 levels are strongly associated with symptom severity, possibly through nociceptor sensitization via inflammatory mediators (36).

### 4.3 NGF and symptom severity

NGF demonstrated a remarkably strong positive correlation with OSDI (*r* = 0.901, *P* < 0.001) and CFS (*r* = 0.701, *P* < 0.001). NGF is a neurotrophin that promotes neuronal growth and differentiation, but also acts as a pro-inflammatory mediator. On the ocular surface, NGF is released under stress conditions and can induce nerve sprouting and sensitization of nociceptors (37). A comprehensive 2025 review by Kocabora et al. consolidated evidence that NGF is involved in both the pathogenesis and potential treatment of DED, noting that recombinant human NGF (cenegermin) has been approved for neurotrophic keratopathy (21).

Lambiase et al. first reported that tear NGF levels are significantly increased in DED and directly correlate with disease severity, particularly with conjunctival hyperemia and fluorescein staining (20). Our findings corroborate and extend these results, showing that the NGF-OSDI correlation is one of the strongest among all tear mediators studied. The lack of correlation between NGF and individual symptoms (other than through OSDI) may indicate that NGF reflects global disease burden rather than specific symptom types. A phase IIa study by Sacchetti et al. showed that topical recombinant human NGF improved symptoms and signs of DED, highlighting the therapeutic relevance of NGF signaling (38).

### 4.4 Substance P and CGRP

SP showed strong positive correlations with OSDI (CC = 0.894) and CFS (CC = 0.711), and was also positively correlated with blurred vision. CGRP, in contrast, showed negative correlations with OSDI (CC = −0.697) and CFS (CC = −0.571). These findings are consistent with the known roles of these neuropeptides. SP is a tachykinin that promotes neurogenic inflammation, plasma extravasation, and nociceptor sensitization (39). In DED, higher SP levels have been linked to ocular discomfort, particularly in patients with more severe symptoms than clinical signs (24, 40).

A recent 2025 longitudinal study by Valencia-Sandonís et al. evaluated 20 mediators and SP in DED patients over at least two years of follow-up. They found that while corneal dendritic cell density decreased over time, SP tear levels actually increased, suggesting persistent neurogenic inflammation even as other inflammatory markers improved (19). This finding is particularly relevant to our cross-sectional results, as it suggests that SP elevation may be a stable feature of DED pathophysiology rather than a transient inflammatory response.

CGRP has more complex roles. While some studies report decreased CGRP in DED (20, 41), others have found that CGRP promotes corneal epithelial proliferation and wound healing (42). The negative correlation with OSDI and CFS observed here suggests that lower CGRP levels are associated with more severe disease, possibly due to impaired neuroprotective mechanisms.

### 4.5 IL-6 and sensory symptoms

IL-6 correlated with multiple sensory symptoms including foreign body sensation, epiphora, itching, and secretion, as well as with OSDI and CFS. IL-6 is a pleiotropic mediator involved in both pro-inflammatory and regulatory functions. A 2024 meta-analysis by Li et al. (from Peking University Third Hospital, the same institution as our study) examined tear mediator levels in Sjögren's syndrome-related DED and found significantly elevated IL-6 levels compared to healthy controls (12). A 2025 study by Chen et al. used latent profile analysis in the DREAM study cohort to identify DED subgroups based on tear mediator profiles, finding that one subgroup (*n* = 23) had significantly higher IL-6 and IL-8 levels, suggesting distinct inflammatory phenotypes that may respond differently to treatment (13). The associations with specific symptoms such as foreign body sensation and itching suggest that IL-6 may contribute to sensory disturbances beyond general inflammation, possibly through direct effects on corneal nerve terminals or through secondary release of other nociceptive mediators (43).

### 4.6 Corneal nerve parameters and tear mediators: a neuro-immune interface

An additional novel finding of this study is the significant correlation between corneal nerve morphology and tear mediator profiles. Specifically, microneuroma count was positively correlated with pro-inflammatory mediators (IL-6, SP, MMP-9) and negatively correlated with the analgesic peptide β-endorphin. Conversely, corneal nerve length was positively correlated with β-endorphin.

Microneuromas are bead-like terminal enlargements of corneal subbasal nerves observed on IVCM, representing aberrant nerve regeneration following injury (44, 45). In DED, chronic ocular surface inflammation and hyperosmolarity can damage corneal nerves, leading to altered regeneration patterns including microneuroma formation (32, 46). Our finding that microneuroma count correlates with elevated IL-6, SP, and MMP-9 suggests that neurogenic inflammation and microneuroma formation may be mutually reinforcing. Damaged nerves release SP and other neuropeptides, which promote inflammation; inflammatory mediators in turn may further impair nerve regeneration, leading to more microneuromas (17, 36). This positive feedback loop may contribute to the persistence of neuropathic ocular pain even after resolution of initial ocular surface damage (22).

The negative correlation between microneuroma count and β-endorphin, together with the positive correlation between nerve length and β-endorphin, suggests that β-endorphin levels are associated with neuroprotective or neurotrophic features in DED. β-endorphin is not only an analgesic but also promotes neuronal survival and axonal growth in animal models (31). Patients with higher β-endorphin levels may have better corneal nerve preservation (longer nerves) and fewer aberrant regenerative structures (microneuromas), which could explain why they report less pain. Conversely, patients with low β-endorphin may be more susceptible to nerve damage and microneuroma formation, leading to neuropathic pain (22).

Clinically, these findings suggest that IVCM assessment of microneuromas, combined with tear β-endorphin measurement, could identify DED patients with a neuropathic pain phenotype who may benefit from neuroprotective or analgesic therapies. Furthermore, interventions that increase β-endorphin, such as transcutaneous electrical nerve stimulation (TENS), acupuncture (22, 26), might simultaneously relieve pain and promote corneal nerve regeneration.

### 4.7 Comparison with previous IVCM and tear mediator studies

Our finding of a positive correlation between CNFL and tear β-endorphin (*r* = 0.722, *P* = 0.030) is, to our knowledge, the first report of this specific association in DED. Previous studies have measured tear β-endorphin in DED patients but did not analyze its correlation with corneal nerve parameters. Zhao et al. (23) reported that patients with chronic ocular pain after FS-LASIK had lower corneal nerve density (*P* = 0.043) and higher tear levels of multiple neuropeptides, including β-endorphin, but did not examine the correlation between β-endorphin and specific nerve parameters. Yang et al. (24) measured tear β-endorphin in DED patients but did not report correlations with IVCM parameters. Our study extends these findings by demonstrating a direct statistical association between endogenous analgesic peptide levels and corneal nerve preservation.

Markoulli et al. (47) examined the relationship between corneal nerve morphology and tear inflammatory mediators in healthy individuals and found that IL-6 correlated negatively with CNFW (average) (*r* = −0.49, *P* = 0.05) and CNFL (max) (*r* = −0.50, *P* = 0.04), while MMP-9 did not correlate with any corneal nerve parameters. In our DED cohort, we did not observe significant correlations between IL-6 or MMP-9 and CNFL. This discrepancy may reflect differences in study populations (healthy individuals vs. DED patients), disease-related inflammatory burden, or the fact that our analysis used averaged CNFL values rather than maximum values.

Regarding microneuromas, our finding that microneuroma count correlates with multiple pro-inflammatory mediators (IL-6, SP, MMP-9) and negatively with β-endorphin is consistent with and extends the work of D'Souza et al. (46), who reported that patients with symptom-sign discordance had a significantly higher proportion of microneuroma-like structures and elevated tear IL-17A levels. Unlike their study, our data also identify associations with SP, MMP-9, and β-endorphin, highlighting additional neuro-immune pathways that may warrant further investigation.

Previous studies have demonstrated that substance P plays a dual role in corneal physiology and pathology, with implications for neurogenic inflammation and wound healing (48). Additionally, CGRP released by corneal sensory nerves has been shown to mediate tear secretion of the lacrimal gland (49). Our correlations between SP and CFS (*r* = 0.711) and between CGRP and CFS (*r* = −0.571) are consistent with these findings, supporting the notion that SP promotes neurogenic inflammation while CGRP may serve a neuroprotective or tear-regulatory function.

We acknowledge that comparisons across studies are limited by differences in DED subtypes, disease severity, tear collection methods, IVCM image analysis software (e.g., ACCMetrics vs. ImageJ vs. NeuronJ), and the specific mediators measured. Our cohort consisted of moderate-to-severe DED patients (mean OSDI 48.85), which may differ from studies including milder cases or specific subtypes such as post-refractive surgery or Sjögren's syndrome-related DED. These methodological differences should be considered when interpreting the present findings in the context of the existing literature.

### 4.8 Implications for symptom-sign discordance

One of the most striking findings of this study is that none of the six tear mediators correlated with TMH or TBUT, whereas all except β-endorphin correlated with CFS. This correlative pattern offers a potential framework for understanding the well-recognized symptom-sign discordance in DED (8, 46). Recent advances in artificial intelligence have enabled automated quantification of meibomian gland morphology, such as meibomian gland density, which has been shown to correlate with MGD severity and may provide complementary information beyond traditional tear function tests (50).While traditional clinical signs of tear quantity (TMH) and stability (TBUT) may not capture the inflammatory and neuropathic processes driving symptoms, tear mediators, particularly those reflecting epithelial damage (MMP-9, NGF, SP, CGRP), showed strong associations with patient-reported outcomes. This aligns with DEWS III's emphasis that DED is “always symptomatic” and that patient-reported symptoms are essential for diagnosis (2). These findings suggest that tear mediator profiling warrants further investigation as a complementary diagnostic tool, especially for patients whose symptoms are disproportionate to clinical signs.

A 2022 study by D'Souza et al. examined corneal nerve morphology features and tear molecular profiles in patients with discordance between clinical signs and discomfort, finding that patients with severe symptoms but mild signs had distinct patterns of corneal nerve abnormalities and mediator elevations (46). Our results complemented these findings by specifically linking individual mediators to particular symptoms and to CFS, a key sign of ocular surface damage.

### 4.9 Clinical implications

The results of this study have several clinical implications. First, tear β-endorphin measurement could potentially identify DED patients with significant neuropathic pain components who may benefit from analgesic therapies targeting endogenous opioid pathways, such as TENS or acupuncture (22). Second, the very strong correlation between MMP-9 and OSDI supports the clinical utility of point-of-care MMP-9 testing (e.g., InflammaDry) not only for diagnosis but also for monitoring symptom severity, especially given the 2025 evidence that clinical phenotypes can predict positive test results (16). Third, the distinct correlation patterns of different tear mediators with specific symptoms suggest that personalized treatment approaches could be guided by individual mediator profiles—for example, patients with high SP might benefit from neurokinin-1 receptor antagonists, while those with low CGRP might respond to CGRP agonists (42). However, such approaches require validation in prospective interventional trials. Fourth, the DEWS III subclassification system provides a framework for integrating mediator profiling into clinical practice (2).

### 4.10 Strengths and limitations

The major strengths of this study include the relatively large sample size (101 patients), the use of a comprehensive 10-symptom questionnaire that captures specific symptom types, the simultaneous measurement of six biologically relevant mediators using sensitive Luminex technology, and the inclusion of β-endorphin, which has rarely been examined in DED tears. Several limitations should be acknowledged. First, the cross-sectional design precludes causal inference; our findings represent statistical associations rather than causal relationships. Second, the absence of a healthy control group prevents us from determining whether the observed mediator levels are abnormal relative to normative values; our findings should therefore be interpreted as associations within a DED population rather than as absolute elevations. Third, although we averaged bilateral ocular parameters to obtain patient-level data and minimize inter-eye correlation, this approach does not fully substitute for statistical methods that account for paired data. Fourth, tear mediator levels may be influenced by multiple factors including age, sex, medication use, DED duration, and environmental conditions, which were not adjusted for due to the exploratory nature and sample size. Fifth, our 10-symptom questionnaire, while capturing a broader range of symptoms than OSDI alone, has not undergone formal psychometric validation; we acknowledge that its use as an independent continuous outcome measure requires further validation in future studies. Sixth, the study population was drawn from a single center in China, which may limit generalizability to other populations. Seventh, while tear collection using microcapillary tubes minimized reflex tearing, we cannot entirely exclude the possibility of stimulus-induced dilution. Eighth, formal repeatability analyses for IVCM nerve parameters and systematic validation parameters (e.g., lower limits of detection, coefficients of variation) for the custom Luminex multiplex configuration were not performed, which may limit comparability with studies using fully validated protocols. Nonetheless, all IVCM quantifications were performed by a single experienced examiner following a predefined protocol, and all Luminex samples were processed under uniform conditions with internal quality controls, supporting the internal validity of our within-cohort comparisons.

### 4.11 Future directions

Future studies should: (1) conduct longitudinal investigations to establish causality between mediator changes and symptom evolution, building on the 2025 Valencia-Sandonís framework (19); (2) include healthy control groups to establish normative reference ranges; (3) explore whether mediator-guided treatment selection improves clinical outcomes; (4) investigate the potential of mediator ratios (e.g., pro-inflammatory to anti-inflammatory ratios) as more robust biomarkers, as suggested by recent DREAM study analyses (6, 51); (5) examine whether tear mediator profiles can distinguish between different DED subtypes as defined by DEWS III, such as tear film deficiencies versus neural dysfunction (2); and (6) integrate tear mediator profiling with artificial intelligence-assisted analysis of multimodal ocular surface data—including meibography, corneal nerve imaging, and clinical parameters—to develop comprehensive, individualized disease characterization models that may ultimately inform targeted therapeutic strategies. The rapid growth of AI applications in ophthalmic imaging provides a strong rationale for such integrative approaches (52).

## 5 Conclusions

This cross-sectional study demonstrates that tear mediator levels are significantly associated with specific ocular symptoms, corneal epithelial damage (CFS), and corneal nerve morphology in DED.β-endorphin shows a strong negative correlation with ocular pain and a positive correlation with corneal nerve length, while microneuroma count correlates positively with pro-inflammatory mediators (IL-6, SP, MMP-9) and negatively with β-endorphin. MMP-9, NGF, and SP show very strong positive correlations with OSDI. None of the tear mediators correlated with TMH or TBUT, suggesting that tear mediators may better reflect the inflammatory and neuropathic mechanisms underlying symptoms and epithelial damage rather than tear quantity or stability. The integration of tear mediator profiling with IVCM assessment of corneal nerves may offer further insight into DED pathophysiology, but this approach requires prospective validation. Future hypothesis-driven, longitudinal studies are needed before mediator profiling can be considered for personalized treatment guidance, particularly for patients with neuropathic ocular pain.

## Data Availability

The original contributions presented in the study are included in the article/Supplementary Material, further inquiries can be directed to the corresponding authors.
