# Supplementary material for: Associations of tear mediators with ocular surface symptoms, signs, and corneal nerve morphology in dry eye disease: a cross-sectional study
Source: Front Med (Lausanne). 2026 Sep 4;13:1929287. doi: 10.3389/fmed.2026.1929287 (PMC13585605; doi:10.3389/fmed.2026.1929287)
Supplement: Supplementary file 5 [file Table5.doc]

Supplementary files. S5
